# Neglected congenital muscular torticollis: A case report

**DOI:** 10.1016/j.amsu.2022.104787

**Published:** 2022-09-29

**Authors:** Irsan Abubakar, Oji Z. Saputra, Diaz Novera

**Affiliations:** a Division of Orthopaedic Surgery and Traumatology, Department of Surgery, Faculty of Medicine, Universitas Syiah Kuala, Zainoel Abidin General Hospital, Banda Aceh, Indonesia; b Division of Orthopaedic Surgery and Traumatology, Department of Surgery, Zainoel Abidin General Hospital, Banda Aceh, Indonesia

**Keywords:** Congenital muscular torticollis, Torticollis, Sternocleidomastoid muscle, Bipolar sternocleidomastoid muscle release, Bipolar tenotomy

## Abstract

**Introduction:**

Congenital muscular torticollis (CMT) is identified as a thickening and/or stiffening of one side of the sternocleidomastoid muscle (SCM) due to muscle fibrosis. This condition results in shortening of SCM and constricted neck motion.

**Case presentation:**

A four-year-old girl came with neck muscle stiffness, tilted head to the left, and chin facing to the right presenting since birth. She was diagnosed with CMT at birth. The patient was born via spontaneous vacuum-assisted vaginal delivery. At three years old, the patient did brief conservative treatment. This patient was planned for unilateral sternocleidomastoid muscle release via bipolar tenotomy. Twelve months after the surgery, there were no complications or recurrence observed.

**Discussion:**

The etiology of CMT remains unknown to date, but recent studies suggest that early treatment of CMT produce better prognosis. The initial treatment for CMT is regular muscle stretching (physiotherapy), as well as education to the child's caregivers about the environmental changes and the child's posture. If the initial attempt fails, surgical intervention is needed.

**Conclusion:**

Early detection and early physiotherapy treatment will lead to minimize the risk of surgery. However, for cases that fail conservative therapy or neglected cases, it is recommended to carry out operative therapy to improve quality of life later.

## Highlights

• Childbirth process and trauma at birth were not the main cause of CMT.
• SCM tumors resolve spontaneously during the first year of life with minimum residual defects.
• Patient's age and history of failure in conservative therapy are considerations for surgery.

## 1 Introduction

Torticollis, most frequently defined as congenital muscular torticollis (CMT), is often found in children and accounts for 3.9% of total pediatric patient cases. CMT also becomes the third most frequent congenital pathologies after congenital hip dislocation and clubfoot [1]. CMT is identified as a thickening and/or stiffening of one side of the SCM due to muscle fibrosis. This condition results in shortening of SCM and constricted neck motion [2,3]. It is assumed that early treatment of CMT produce better prognosis. The initial treatment of CMT begins with conservative therapy consisting of educating the children's caregivers concerning environmental changes and child position [4,5]. We report a case of a 4-year-old girl with neglected CMT that has satisfactory outcome after undergoing bipolar SCM muscle release. This case report has been reported in line with the SCARE Criteria [6].

## 2 Case presentation

The patient was referred to our outpatient clinic presenting with the chief complaint of neck muscle stiffness, tilted head to the left, and chin facing to the right presenting since birth. She was diagnosed with CMT at birth. Pain, blurry vision and other neurological disorders were denied.

From history taking, it was known that The patient was born via spontaneous vacuum-assisted vaginal delivery due to prolonged second-stage labor. She did not have any history of prolonged infection as well as surgical history in the head and neck area. No other congenital abnormalities were found.

At three years old, the patient did brief conservative treatment with a physiotherapist for one month to stretch the sternocleidomastoid muscle but the treatment was stopped by her parents due to the distance between health facilities and the patient's house being quite far away and it was inconvenient to go there routinely. Her medical and surgical history was unremarkable.

On physical examination, her face was slightly asymmetry, her head turned toward the left side and her chin pointed to the right side. We found a shortened, prominent and cord-like left sternocleidomastoid muscle. There was limited rotation of the neck to the right side. There was no abnormal posturing of other body parts.

**Fig. 1:**
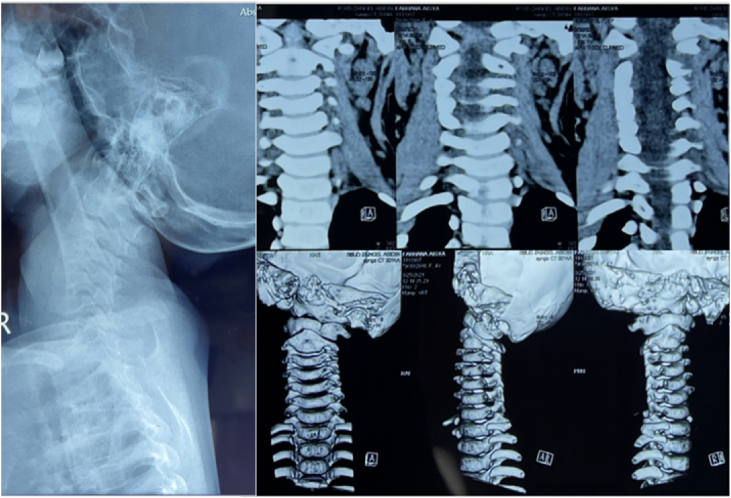
Cervical plain radiograph and cervical spine CT-Scan.

Pre-operative cervical plain radiograph and CT-Scan shows no signs of maldevelopment or subluxation of the cervical vertebrae (Fig. 1). The patient was planned for unilateral SCM muscle release via bipolar tenotomy under general anesthesia.

**Fig. 2:**
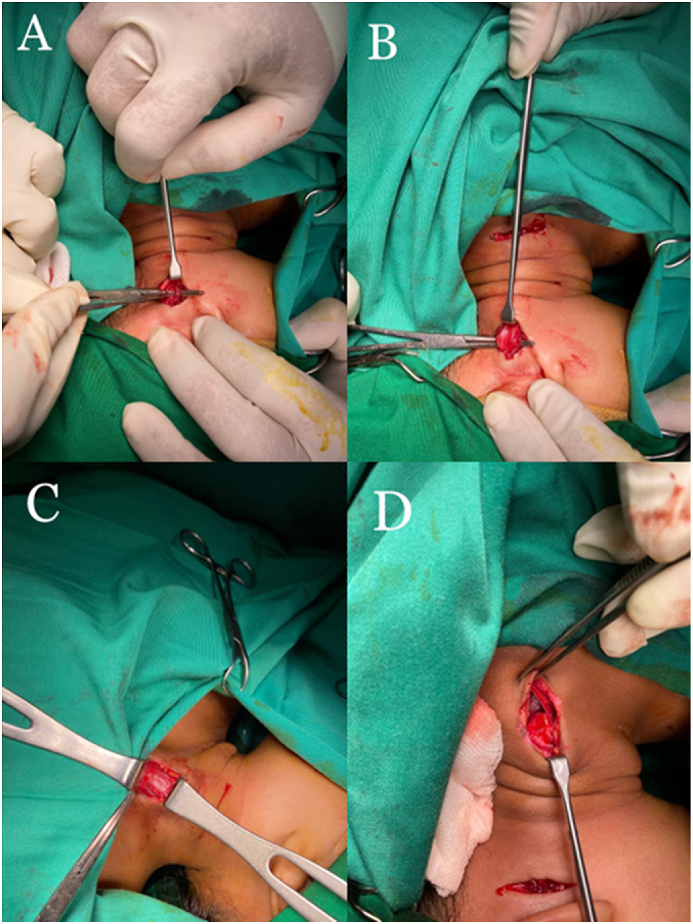
Intraoperative documentation. (A) The incision site was located below the mastoid region, the overlying greater auricular nerve was recognized and preserved during dissection. (B) The attachment of SCM muscle to mastoid bone was spotted and entirely released from its bony attachment. (C) Both the clavicular and sternal heads of the sternocleidomastoid muscle were distinguished and then dissected directly along with the deep fascia. (D) The sternal tip was sutured to the clavicular cut end in an oblique line to achieve muscle lengthening.

The operation was performed by a general Orthopaedic surgeon in tertiary referral hospital. An incision was placed 2 cm below the mastoid on the left side. We spotted the attachment of SCM muscle to mastoid bone and entirely released from its bony attachment, securing the underlying crucial structures and overlying the greater auricular nerve. Another incision was made transversely about 2.5 cm above the clavicle-sternum junction on the left side and the platysma was released as well. Two SCM heads were distinguished and then dissected directly along with the deep fascia. The muscle might retract (Fig. 2). The sternal tip was sutured to the clavicular cut end in an oblique line using a 4/0 vicryl suture. After bleeding was controlled, the skin is closed by 4/0 prolene. We ensure that complete neck extension was obtained without any tension.

**Fig. 3:**
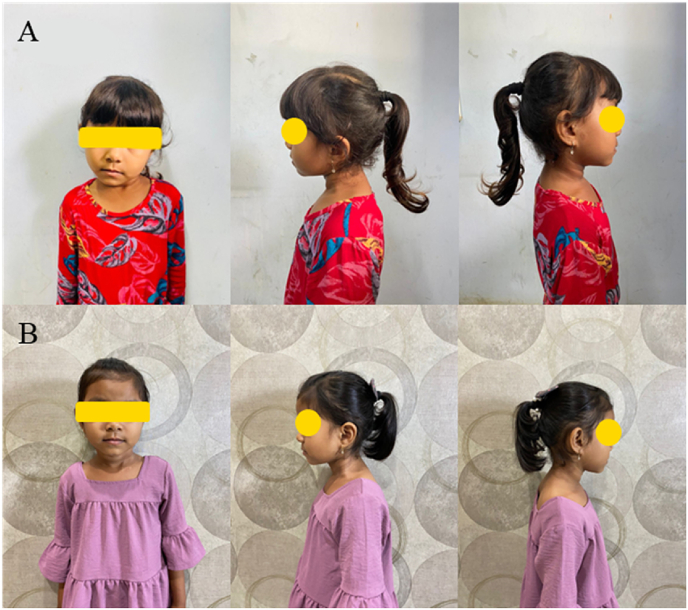
(A) Preoperative clinical photograph showing the child with congenital muscular torticollis affecting the left sternocleidomastoid muscle. (B) At 12 months postoperative, the patient's neck becomes straight with a normal range of neck movement.

Until one week after the surgery, the patient was immobilized in the corrected position using a plaster of Paris. Twelve months postoperative, there was no complications or recurrence observed (Fig. 3).

## 3 Discussion

Torticollis is a deformity of the head and can be caused by congenital or acquired abnormalities. Acquired torticollis can be caused by trauma, past-infection process, or malignancy. While congenital muscular torticollis (CMT) is a deformity of the neck and head that can be detected at birth or at any time before birth caused by unilateral fibrosis and shortening of the sternocleidomastoid muscle. Torticollis is also known as a twisted neck [2]. The shortening of sternocleidomastoid muscle results in the pulling of the mastoid process toward the sternoclavicular joint [7].

The etiology of CMT remains unknown. Some theories suggest that molding during pregnancy is the cause of the deformity [8]. Leading theories behind SCM muscle impairment in CMT include intrauterine crowding, muscle trauma during a difficult labor, soft-tissue compression resulting in compartment syndrome and congenital abnormalities of soft-tissue differentiation within the SCM muscle [9]. Nevertheless, Hardgrip et al. reported that the childbirth process and trauma at birth were not the main cause of CMT [10].

Several complications can arise from neglected CMT, one of which is cervical spine alterations. Husein et al. found that the onset of deformable changes in the cervical spine begins as early as 8 months of age, and the severity of the deformity increases with age and SCM tightness [11]. Infants with CMT must receive early intervention to minimize these secondary impairments.

Approximately 50–70% of SCM tumors resolve spontaneously during the first year of life with minimal residual defects [12]. However, SCM tumors still requires close monitoring at this stage.

The initial treatment for CMT is conservative. It is recommended to perform regular stretching exercises consisting of flexion-extension, lateral bending, and rotation of the neck. Sets of 15 stretches, which consist of holding the stretch for 1 s followed by 10-s rest in between and performed three times per week was found effective to improve range of motion [13]. The choice of surgery in this patient was due to failure of conservative treatment and the patient's age.

There are other options of surgical procedure for neglected CMT namely unipolar SCM muscle lengthening, bipolar SCM muscle lengthening, Z lengthening, or radical resection of the SCM [8]. Recurrence is the main risk postoperatively but Chotigavanichaya et al. found that sex, side of deformity, type of surgery, and age at the time of surgery has no correlation with recurrence of the deformity [14].

## 4 Conclusion

Early detection and physiotherapy treatment of CMT will lead to satisfactory results and minimize the risk of surgical intervention. However, failed conservative treatment or neglected cases prompts for surgical intervention to improve the patient's quality of life.

## Ethical approval

None.

## Please state any sources of funding for your research

We declare no funding for this study.

## Author statement contribution

All authors contributed equally, have read and approved the final manuscript.

## Please state any conflicts of interest

We declare no conflicts of interest for this study.

## Guarantor

Irsan Abubakar, MD Backspace

## Consent

Written informed consent was obtained from the patient for publication of this case report and accompanying images. A copy of the written consent is available for review by the Editor-in-Chief of this journal on request.

## Provenance and peer review

Not commissioned, externally peer-reviewed.
